# “Up” or “down” that makes the difference. How giant honeybees (*Apis dorsata*) see the world

**DOI:** 10.1371/journal.pone.0185325

**Published:** 2017-11-30

**Authors:** Nikolaus Koeniger, Christoph Kurze, Mananya Phiancharoen, Gudrun Koeniger

**Affiliations:** 1 Molecular Ecology, Institute of Biology/Zoology, Martin-Luther-University Halle-Wittenberg, Halle an der Saale, Germany; 2 Center for Infectious Disease Dynamics, Penn State University, University Park, PA, United States of America; 3 King Mongkut’s University of Technology Thonburi, Bangkok, Thailand; Universidade de Sao Paulo Faculdade de Filosofia Ciencias e Letras de Ribeirao Preto, BRAZIL

## Abstract

*A*. *dorsata* builds its large exposed comb high in trees or under ledges of high rocks. The “open” nest of *A*. *dorsata*, shielded (only!) by multiple layers of bees, is highly vulnerable to any kind of direct contact or close range attacks from predators. Therefore, guard bees of the outer layer of *A*. *dorsata’s* nest monitor the vicinity for possible hazards and an effective risk assessment is required. Guard bees, however, are frequently exposed to different objects like leaves, twigs and other tree litter passing the nest from above and falling to the ground. Thus, downward movement of objects past the nest might be used by *A*. *dorsata* to classify these visual stimuli near the nest as “harmless”. To test the effect of movement direction on defensive responses, we used circular black discs that were moved down or up in front of colonies and recorded the number of guard bees flying towards the disc. The size of the disc (diameter from 8 cm to 50 cm) had an effect on the number of guard bees responding, the bigger the plate the more bees started from the nest. The direction of a disc’s movement had a dramatic effect on the attraction. We found a significantly higher number of attacks, when discs were moved upwards compared to downward movements (GLMM (estimate ± s.e.) 1.872 ± 0.149, P < 0.001). Our results demonstrate for the first time that the vertical direction of movement of an object can be important for releasing defensive behaviour. Upward movement of dark objects near the colony might be an innate releaser of attack flights. At the same time, downward movement is perceived as a “harmless” stimulus.

## Introduction

**Fig 1:**
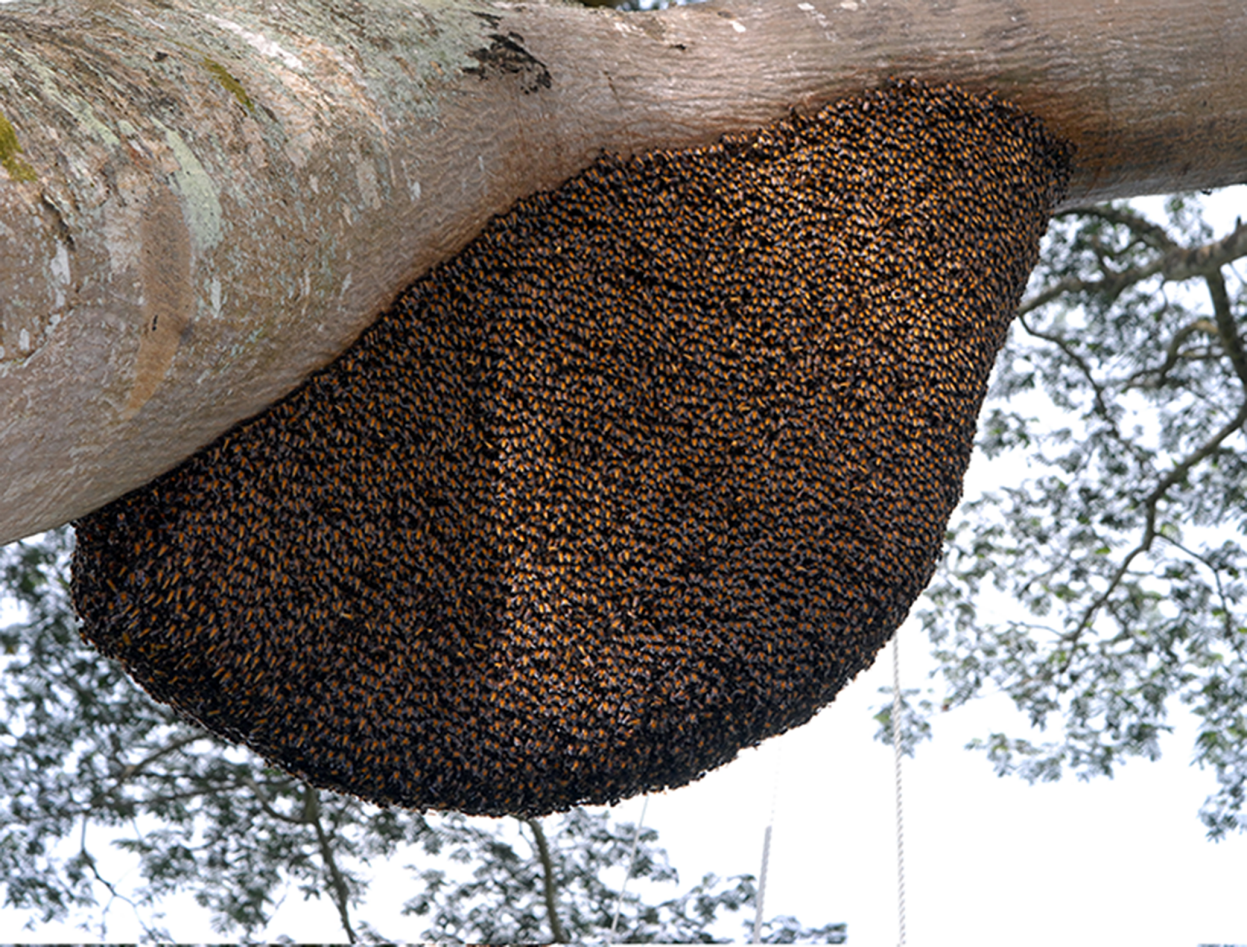
*A*. *dorsata* colony nests high up under a thick horizontal branch.

*Apis dorsata* nests in the open and builds its single comb below thick relatively horizontal branches high up in trees or under ledges of high rocks (Fig 1). The biomass of an *A*. *dorsata* nest comprises of more than 5 kg protein (bees, brood and pollen storage) and significant amounts of carbohydrate (honey).

So *A*. *dorsata* nests are rewarding targets for predators and because predation is a major force in shaping the behaviour of animals, the exposed position of *A*. *dorsata* colonies suggests that they have very effective defence behaviour. Indeed, several very impressive samples of *A*. *dorsata*’s successful colony defence have been reported [1–5].

**Fig 2:**
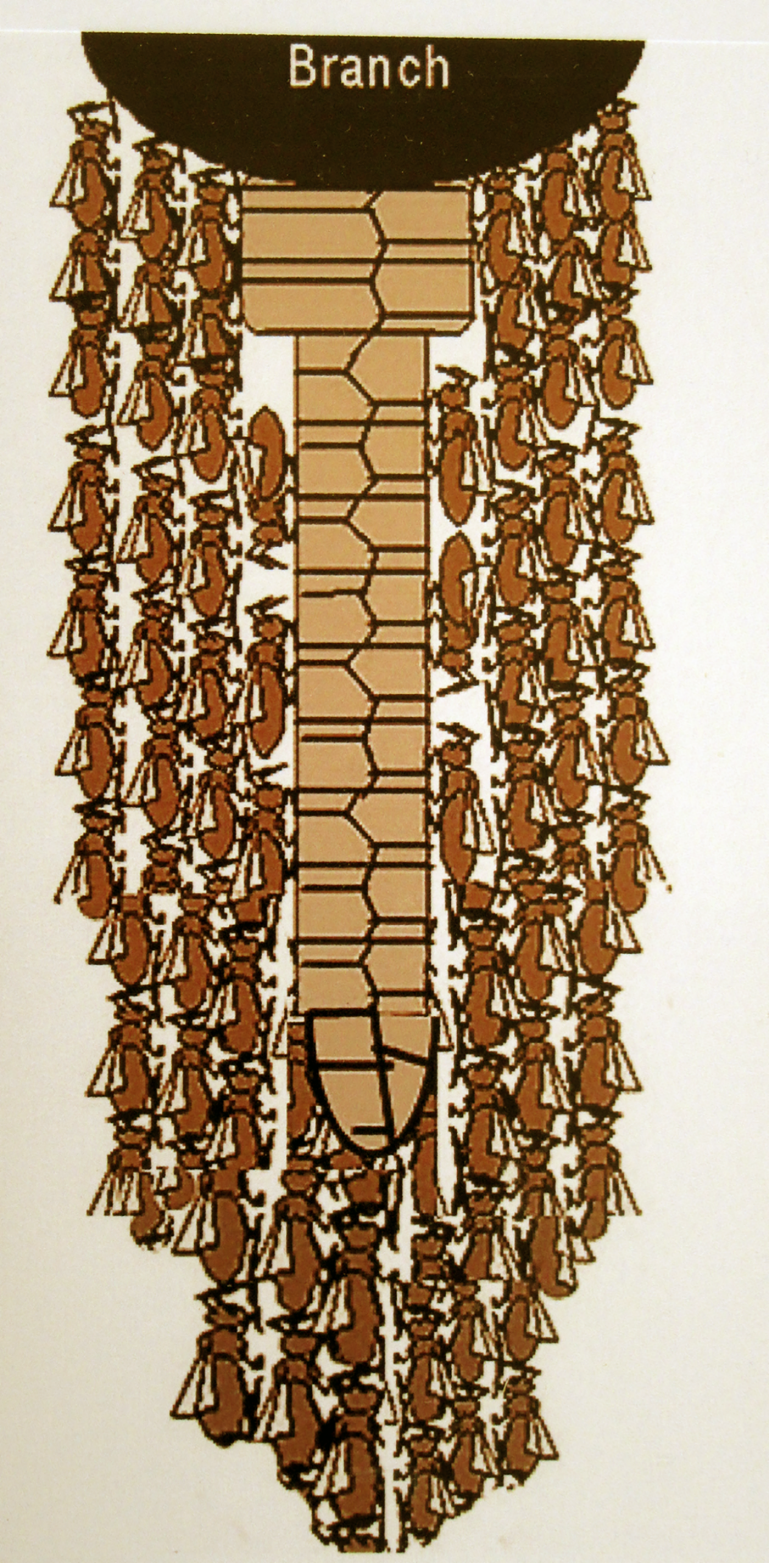
“Envelope” of bees shielding the comb (schematic cross section).

The single comb of *A*. *dorsata* is covered by worker bees. They hang with their heads up and abdomens down like a curtain from the branch or the support to which the comb is attached. Beneath the comb, the curtains on each side of the comb merge together totally enclosing it in an envelope consisting of multiple layers of worker bees (Fig 2). About 60 to 80% of the total worker population participates regularly in this envelope covering the comb containing brood, pollen and honey stores [6].

The number of predators threatening a prey animal may be very large [7] and the range of potential predators or honey robbers of *A*. *dorsata* colonies encompasses bears, buzzards, smaller birds, climbing reptiles, predatory insects (*Vespa spec*., *Mantidae*, ants etc.) honey stealing butterflies or minute fruit flies. The sheer numbers of those species (predators and intruders) seem to preclude any species specific predator recognition [8]. Instead, to react adequately and successfully, *A*. *dorsata* must possess the ability to generalize predator recognition. Generalization of predator recognition is well documented for vertebrates [9–11] but has not been considered in the context of colony defence of *Apis*.

A major general cue for threats is detection through the visual sense, predominantly the detection of movement. Butterflies and other flying insects approaching the nest are repelled by “shimmering”, a specific movement of bees in the curtain which prevents the touch down of those insects on the bee’s nest [12–13]. Approaching birds, for example attacking honey buzzards (*Pernis ptilorhynchus torquatus* Lesseon 1831), release a sudden transformation of the curtain and bees build chains at the lower rim of the comb for defence against those predators that try to break off parts of the combs with their claws from below [5]. Yet another defensive reaction by the bees is the production of hissing sounds released by mechanical stimuli that are caused by mammals climbing up to the nest. When this sound does not ward off the intruder, it is followed by mass sting flights of guard bees [14]. The diversity of colony defensive reactions of *A*. *dorsata* surpasses the array of colony defences of *A*. *mellifera* and the other cavity dwelling *Apis* species by far [5]. For a general and consistent review of colony defence in *Apis* species including *A*. *dorsata* see [12] and for a detailed analysis of several behaviours of *A*. *dorsata’s* colony defence with a focus on the communication within the colony see [13].

In contrast to cavity dwelling *Apis* species, the “open” comb is particularly vulnerable to any kind of direct contact or close range attacks by predators [15]. Early detection of any danger at an appropriate distance is essential. Therefore, the bees of the outer layer of *A*. *dorsata’s* envelope must monitor the vicinity for possible hazards in all directions. Effective risk assessment is required to balance the costs of defence against other colony activities such as brood rearing and foraging.

Any sophisticated system of enemy detection, however, leaves the bees with a fundamental dilemma: Many movements near the nest are not related to potential threats. Guard bees on the outside of the curtain are frequently exposed to different objects like leaves, twigs, dead branches, flowers or pieces of bark passing the nest from above and falling to the ground. Casual observations in trees of *Falcataria moluccana* at ARS Tenom of colonies *A*. *dorsata* at a height of 25 m to 32 m resulted in more than 20 objects/hour falling down within a range of 2 m from the nest. It is not clear how guard bees on the curtain monitoring the vicinity classify such movements near the colony as not requiring a response. The shape, the size and the falling speed differs significantly among dead leaves, pieces of bark or dead branches and the only obvious invariance seems to be the downward direction of movement. Thus downward movements might be utilized by *A*. *dorsata* to classify visual stimuli near the nest as “harmless”.

Alternative to an innate mechanism to minimize alarm reactions could be habituation to downward movements near the nest. Thus, the frequent occurrence of falling objects without any disturbance of the colony might cause desensitization. To test the effect of the direction of movement of objects on defensive responses, we used black circular discs moving either down or up in front of colonies and recorded the number of guard bees flying towards the disc.

## Methods

The experiments were carried out in February and March 2011 at the Agricultural Research Station Tenom (ARS Tenom, Sabah, Malaysia). Field permit was granted by chief officer of ARS Tenom. The experiments were limited to the station's areas. We tested three *A*. *dorsata* colonies with brood and two swarms at their natural nesting sites. The size of the colonies with brood was about 70 cm x 50 cm, that of swarms 50 cm x 40 cm. One colony with brood had built its comb at a height of 5 m under the roof of the Bee Museum. The other two were attached to branches of trees, one in field 21 was at a height 6 m, the other in field 43 at a height of 8 m. The swarms were also on tree branches, one at the natural lake at a height 12 m, and the other in field 25, at a height of 3.50 m. We performed experiments at each bee colony for 3 to 8 days.

For the experiments, we used black wooden discs (plywood 3 mm thick) with a diameter of 50 cm, 25 cm and 8 cm which we fixed to a stick of dry bamboo (length was adjusted to the height of the colony). The discs were moved either up or down in front of the colonies at a distance of 1.5 m (visual angle: 50 cm = 19°. 25 cm = 9°31’ and 8 cm = 3°3’). The amplitude of the movement was 1.6 m, from 40 cm above the upper rim of the comb to 120 cm below it. The speed of the disk was about 1m/sec. After each movement, we waited until the bees had calmed down and had returned to the nest (mostly for about 2 min). When more than 25 bees reacted to the discs, we stopped the experiments at the colony. A period of 1–3 hours was allowed to elapse before the next test series was performed at each colony. We changed the test disc whenever a guard bee touched or landed on a disc.

The number of bees leaving the curtain of the colony in direction to the black disc was counted directly by two independent observers. Whenever both counts matched, the test was considered valid. In all cases—when the number of guard bees leaving the curtain was above 20—we accepted differences of both counts and took the mean value of both counts.

Regularly we observed increased general defence activity during the course of the trials. Stinging behaviour together with effects of *A*. *dorsata’s* alarm pheromones [16] forced us to increase the time interval between tests to permit colonies to get back to normal foraging activity ensuring comparable initial behavioural conditions for subsequent tests.

## Statistics

Statistical analyses and data plotting were performed in R version 3.3.1 [17]. We fitted the number of attacks in a generalized linear mixed model (GLMM) with Quasi-Poisson error distribution using the Automatic Differentiation Model Builder package (*glmmADMB*) in R [18]. This method was chosen to limit the effects of over-dispersion of the data, using *overdisp*.*glmer*, with checking of the residuals. We included “colony” and “disc size” as random factors to account for colony and disc size-specific effects. Our final model only included disc movement direction as a fixed factor (Logan, 2010).

## Results

**Fig 3:**
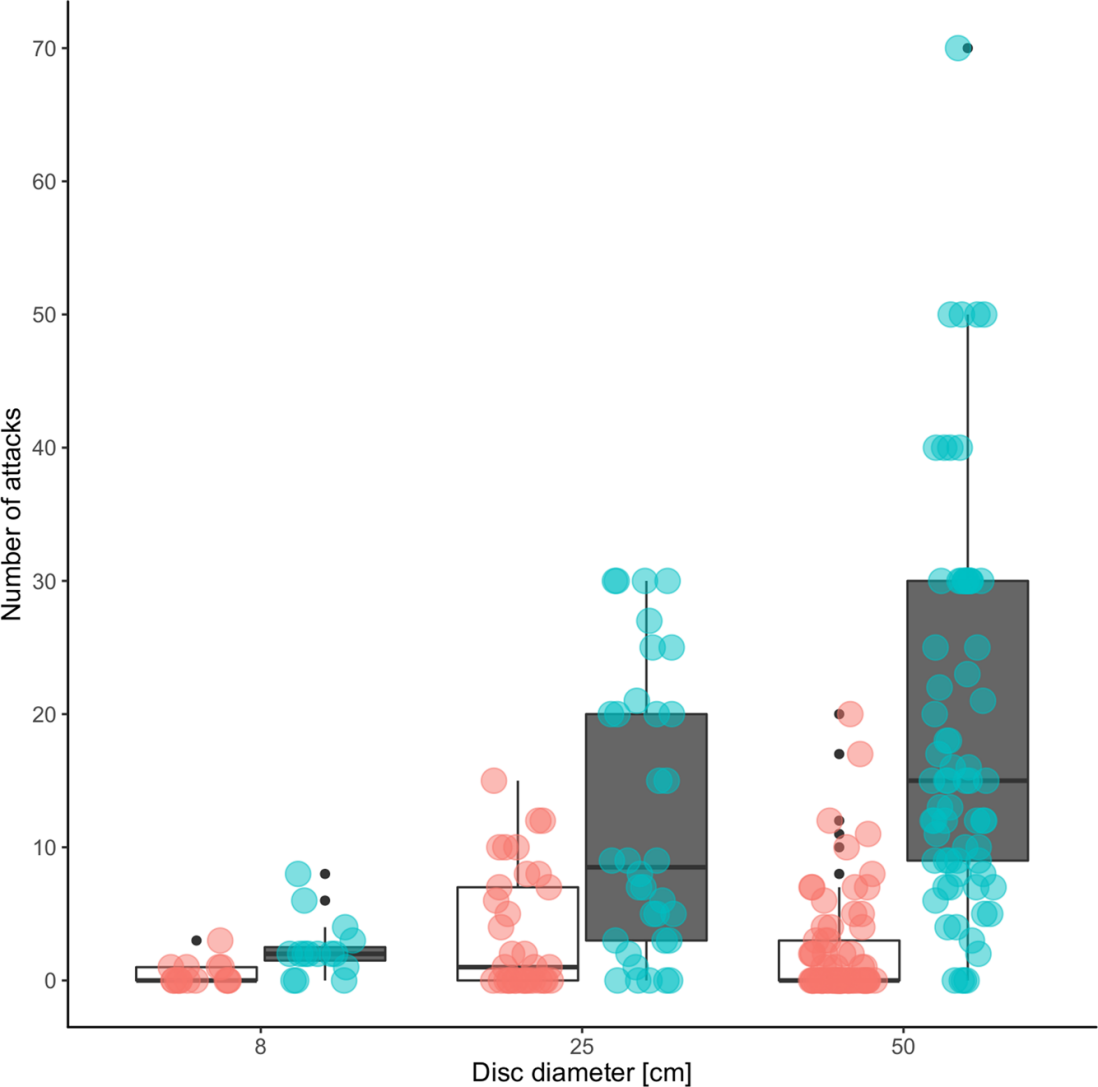
Number of attacking bees in response to up and downwards movement in three different disc sizes (8 cm, 25 cm, 50 cm). Discs were displayed in a 1.5 m distance to the nests. Box plots in white with red scatter plot overlay represent number of attacking bees during downward movement of discs. Box plots in grey with cyan scatter plot overlay represent number of attacking bees during upward movement of discs respectively. The total number of observations were 15 for 8 cm (colony 1 only), 34 for 25 cm (colonies 1 and 3) and 70 for 50 cm (colonies 1, 2, 4 and 5) disc sizes respectively.

At *A*. *dorsata* colony 1 we tested discs with diameters of 8 cm, 25 cm and 50 cm. In all 69 trials, more bees responded after the “up” than after the “down” movement. The median of the large disc (diameter 50 cm) was 9 responses while that of the small disc (diameter 8 cm) had only 2 (Fig 3). The diameter of the disc had an effect on the number of bees, the bigger the plate the more bees responded.

**Fig 4:**
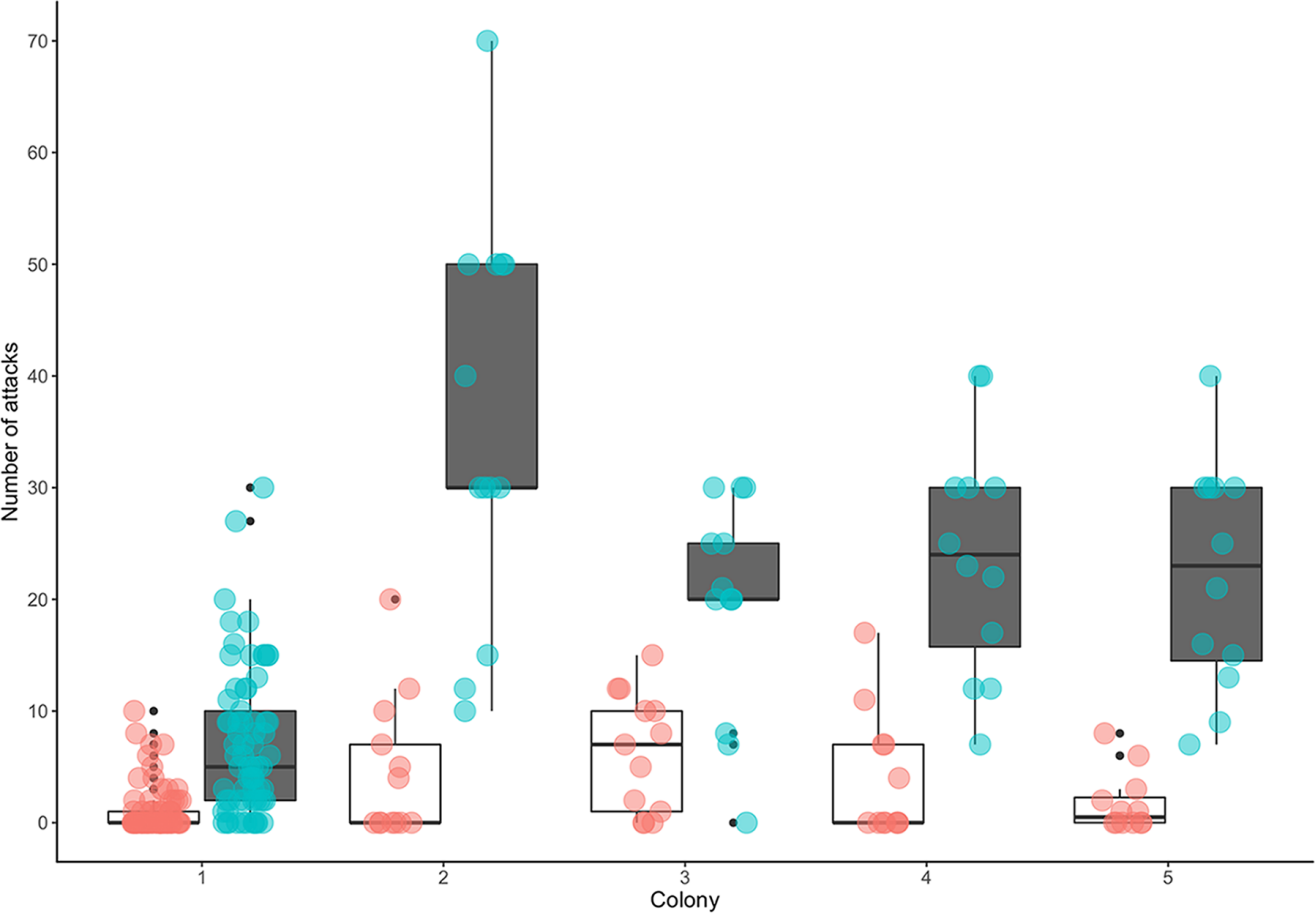
Number of attacking bees for each of the five colonies displayed separately. Discs were displayed in 1.5 m a distance to the nests. Box plots in white with red scatter plot overlay represent number of attacking bees during downward movement of discs. Box plots in grey with cyan scatter plot overlay represent number of attacking bees during upward movement of discs respectively. The total number of observations were 69 for colony 1, 13 for colony 2, 13 for colony 3, 12 for colony 4, 12 for colony 5 respectively.

To expand our tests to additional colonies we used discs with a diameter of 50 cm besides at colony 3 that we tested with discs of 25 cm diameter. A majority of defending guard bees responded to the disc upon the upward movement (medians of responses ranged from 9 to 30). In contrast, the number of bees reacting upon the downward movement was low (medians of responses ranged from 0 to 6. (Fig 4).

We found a significantly higher number of attacks, when discs were moving upwards compared to downwards (GLMM (estimate ± s.e.) 1.872 ± 0.149, *P <* 0.001).

## Discussion

Colony defence of *A*. *dorsata*—as well as other animals—depends on recognition of predators. As mentioned before the great variety of honey thieves and predators might not allow recognition at the species level. Instead, generalization and classification of intruders and predators is a prerequisite condition for successful colony defence. In particular, because of the fragile open comb of *A*. *dorsata* early detection of predators and intruders at an adequate distance is indispensable to facilitate defensive behaviours of guard bees protecting the comb. Therefore, visual cues seem to play a key role. Fast moving objects with distinct horizontal displays typical of flying insects or small birds release “shimmering” and prevent the landing of such intruders on the nest. Furthermore, it was demonstrated that shimmering waves of *A*. *dorsata* guard bees drove away attacking hornets [19]. A more specific defensive behaviour was observed when honey buzzards (*P*. *ptilorhynchus torquatus*) attacked *A*. *dorsata* colonies. Immediately many guard bees left the comb and built chains shielding the comb against attacks from below. Further, clusters of guard bees became airborne and started counter attacking the birds and people under the tree as well. A similar behavioural response was released by horizontal and circular movement of a cardboard dummy in shape and size of a buzzard [5].

Testing the vertical movement of the black disc, we did not observe any change in the pattern of the colony’s curtain or shimmering. Guard bees started from dispersed places in the curtain and flew fast in the direction of the disc. Many of these bees did not touch the disc. Instead, they changed their flight direction shortly before touching the disc and started patrolling the surroundings of the nest. These guard bees attacked and stung people close to the colony. Apparently, the moving discs released a counter attack [20] and stinging behaviour that is a typical defensive response whenever the survival of the colony is at risk. Stinging mammalian predators or birds is “costly” for the colony because the sting remains in the skin of predator and the guard bee will die within a short period. Considering the colony’s behavioural response to the black disc moving upward suggests that this visual cue is construed to be a large hazardous predator as the response increases with disc size. Sun bears (Heliarctos malayanus), humans (honey hunters) and monkeys might release this defensive behaviour.

The nest of *A*. *dorsata* will not require protection against heavy objects falling on it. The thick and robust branches under which colonies are constructed offers protection against such falling objects or intruders coming from above (Fig 1).

The striking difference in response in relation to “up” or “down” movements might be a result of habituation. As mentioned earlier colonies nesting in trees are exposed to many falling objects. Guard bees in the curtain perceive that objects moving downward are not a source of threat to the colony. Certain colonies of *A*. *dorsata*, however, which nest under rock ledges or under eaves or margins of temples and other high buildings are not exposed to falling tree litter. In our experiments colony 1 nested under the roof of the bee museum in Tenom and thus these guard bees were not habituated to falling items. Nevertheless, guard bees from this colony had a comparable preference for attacking the dummy moving up as the guards of the other colonies that were tested. This may indicate that upward movement of dark objects near the colony is an innate releaser for attack flights. At the same time, downward movements are perceived as harmless stimuli. In this way Newton`s law of gravity is instrumental for guard bees of *A*. *dorsata* to distinguish between harmless and dangerous movements near the nest. These results demonstrate for the first time that the vertical direction of movements is an important innate cue for predator recognition in *A*. *dorsata*.

Further experiments with other honeybee species would be of scientific interest. *A*. *dorsata* as a member of the subgenus *Megapis* is supposed to present a medium stage (between Micrapis and cavity dwelling honey bees) in the evolution of the genus [21, 22]. However, its biology as a migratory species and the nest construction below a solid support is not shared by the other species in the free nesting subgenus *Micrapis* (dwarf honey bees) which build their single comb hidden in the undergrowth around a twig or small branch with the honey stores above [23]. Therefore, these colonies are not sheltered against any impact from above like *A*. *dorsata*. The third subgenus *Apis* comprises 5 cavity dwelling species. Their nests are primarily protected by the walls of their nest’s cavity and the colonies are often hidden. Monitoring the surroundings of their nests is much less demanding than for the exposed open nesting *A*. *dorsata*. Cavity dwelling species can focus their guarding at the cavity’s entrance [5]. In spite of a lack of experimental evidence, we hypothesize that the recognition of “harmless” movements near the nest based on their downward direction is a unique behaviour of *Megapis* (*A*. *dorsata* or *A*.*laboriosa*) and guard bees of other subgenera looking out of their nests might see the “world” differently.

## Supporting information

S1 File Mappe1. (XLSX)
